# How Often Are Major Blood-Borne Pathogens Found in Eye Patients? A Serosurvey at an Eye Hospital in Southern China

**DOI:** 10.1371/journal.pone.0073994

**Published:** 2013-09-04

**Authors:** Fang Duan, Qiang Huang, Jingyu Liao, Dajun Pang, Xiaofeng Lin, Kaili Wu

**Affiliations:** State Key Laboratory of Ophthalmology, Zhongshan Ophthalmic Center, Sun Yat-sen University, Guangzhou, China; Zhongshan Ophthalmic Center, China

## Abstract

**Background:**

The hepatitis B virus (HBV), hepatitis C virus (HCV), human immunodeficiency virus (HIV) and treponema pallidum (TP) are blood-borne pathogens. They can lead to nosocomial and occupational infections in health care settings. We aimed to identify the prevalence of and risk factors associated with HBV, HCV, HIV and TP infections among patients with eye diseases at a tertiary eye hospital in Southern China.

**Methods:**

From July 2011 to June 2012, a total of 26,386 blood units were collected from eye patients, including inpatients and the day surgery patients at Zhongshan Ophthalmic Center, one of the biggest eye hospitals in China. Based on the primary diagnoses from this period, the subjects were classified into different ocular disease groups. All blood samples were tested for HBsAg, anti-HCV, anti-HIV and anti-TP.

**Result:**

The overall prevalence of HBV, HCV, TP and HIV was 9.79%, 0.99%, 2.43% and 0.11%, respectively. The prevalence of HBsAg was much lower among patients younger than 20 years compared to other age groups. In addition, the risk of HBsAg was associated with the male gender, ocular trauma and glaucoma. The prevalence of TP increased with age and the prevalence among patients older than 30 was higher than that in patients younger than 20 years.

**Conclusions:**

The prevalence of HBV, HCV, HIV and TP in patients with eye diseases was identified. This information can be utilised to strengthen the health education and implementation of universal safety precautions to prevent the spread of blood-borne pathogens in health care settings.

## Introduction

The transmission of blood-borne pathogens, e.g., the hepatitis B virus (HBV), the hepatitis C virus (HCV), the human immunodeficiency virus (HIV) and treponema pallidum (TP), has been well-documented in health care settings, where these pathogens have been reported to be transmitted from patients to hospital care personnel (HCP), from HCP to patients and from patient to patient. Infections by these pathogens are the most common and important chronic infectious diseases worldwide and remain a large health care burden globally. The World Health Organization estimates that worldwide at least 33 million people are chronically infected with HIV [1], 170 million with HCV [2], 350 million with HBV [3] and approximately 12 million with TP [4]. Furthermore, the most affected populations live in developing countries, where the economy is bad and there is no or low levels of health care. It is well know that of those exposed to these blood-borne and sexually transmitted infections, commercial sex workers, men who have sex with men and intravenous drug users are at the highest risk. More importantly, hospital exposures, including patients receiving injections and/or blood transfusions [5], [6] and HCP subject to occupational exposures and infections [7], [8], are one of the major forms of transmission of these blood-borne pathogens and need to be prevented. In addition, the transmission of these pathogens after a percutaneous exposure also contributes greatly to infections.

Therefore, the World Health Organization recommends universal and quality-controlled screening of all blood donations for HIV, HBV, HCV and TP [9]. Until now, mandatory testing for these pathogens has been an appropriate measure for blood, tissue and organ donations to improve the safety of the medical procedures. In other health care activities, such as elective surgeries and hospitalised patients, testing for blood-borne pathogens based on clinical indications, is recommended and utilised worldwide to reduce the probability of nosocomial and occupational infections. Information of a patient’s blood-borne pathogen status may be relevant in planning surgical procedures and could lead to the utilisation of safer techniques (e.g., extra gloves, sharps protections) for both HCP and patients. Furthermore, if exposure does occur, knowing the patient’s infection status will help infected personnel expedite the acceptance of appropriate post-exposure treatments.

Although a number of studies, which mainly focused on blood donors [10]–[13] and high-risk populations [14]–[17], have monitored the epidemiology of HIV, HBV, HCV and TP, data revealing the epidemiology of these pathogens in a specific group of patients are rare. For example, in an Indian study with 256 patients with chronic renal failure (138 had renal transplant and 118 were on maintenance haemodialysis), 7% were infected with HBV, 46% were infected with HCV [18]. The prevalence of HBV and HCV infection in 155 myeloma patients was reported as 11% and 9%, respectively, in Taiwan [19]. In general, the prevalence of infection is higher among hospitalised patients than in the general population.

Although much attention has focused on preventing transmission by blood transfusions and organ transplants, it is also important to be mindful of other hospital-associated infections such as blood-borne transmissions. More and more cases of healthcare-associated transmissions have being reported in professional literature, government reports and even in the public news [20]–[23]. In addition to transmission by blood and sexual contact, pathogens can be transmitted through other body fluids and mucosal membranes, such as conjunctiva. There have been two reported cases of HCV transmission resulting from a blood splash to the conjunctiva [24], [25]. In addition, HIV genes and proteins were detected in ocular surface cells and tears, even in patients on anti-HIV treatment [26], [27]. Therefore, ophthalmological practitioners have been encouraged to become familiar with the knowledge of the blood-borne pathogen status of their eye patients, which could be helpful in preventing healthcare-associated transmission of these pathogens. Till now, there has been no report on the prevalence of the major blood-borne pathogens in hospitalised eye patients.

In the current study, we assessed the serological prevalence of HIV, HBV, HCV and TP among hospitalised and/or operated eye patients at the Zhongshan Ophthalmic Centre, one of the biggest eye hospitals in China, from July 2011 to June 2012. We obtained a general prevalence for these four pathogens in eye patients and found that there was a higher prevalence for HBV in glaucoma and ocular trauma patients as compared to lens disorder patients.

## Methods

### Study Design

A cross-sectional study was conducted to estimate the prevalence of and risk factors associated with HBV, HCV, TP and HIV among ocular inpatients and surgery patients during 2011 and 2012.

### Study Population and Specimen Collection

From July 2011 to June 2012, a total of 26,386 blood samples from eye patients who were hospitalised and/or operated on for the first time were collected at the Zhongshan Ophthalmic Centre, Sun Yat-sen University. The patients in our study were mainly from ten provinces of southern China and the population of these provinces accounts for roughly a third of the total population of China. Blood samples (3–5 mL) were collected from each patient and were assessed at the hospital’s clinical laboratory. Under the guidelines of the Ministry of Health of China (2000, No.184 and 2009, No.111), routine testing for the four pathogens in inpatients and surgery patients were conducted at the Zhongshan Ophthalmic Center. The Ethical Committee of Zhongshan Ophthalmic Center waived the patients’ consent for the above testing and approved this study.

### Laboratory Testing

The serum specimens were screened for hepatitis B virus surface antigen (HBsAg), anti-HCV, anti-HIV and anti-TP using the Abbott ARCHITECT® i2000SR system and accompanying reagents (Abbott, Illinois, USA). The Diagnostic Kit for Hepatitis B Surface Antigen (6C36), the Diagnostic Kit for Antibody to HIV (4J27), the Diagnostic Kit for Antibody to syphilis TP (8D06) and the Diagnostic Kit for Antibody to Hepatitis C Virus (6C37) were used, respectively. According to the recommended criteria from Abbott, readings over 0.05 U/ml for HBV and greater than 1 S/CO for HBV, HCV and HIV were considered positive. HIV infections were reconfirmed by Western blot analysis at the Centre for Disease Control and Prevention in Guangzhou.

### Data Analysis

The general seroprevalence of the four pathogens in various eye patients was determined. Chi-square test was used to determine the differences among categorical variables (age group, gender and ocular diseases). Odds ratios (ORs) and their corresponding 95% CIs for the association between potential risk factors (age, gender and ocular diseases) and pathogen infection (HBV and TP) were computed by univariable logistic regression.

Based on the primary diagnoses from this period, the subjects were classified into cornea diseases, lens disorders, glaucoma, uveitis, ocular trauma, strabismus, diseases of the vitreous/retina, optic nerve, orbital (except ocular tumour), lacrimal duct, oculoplastics, ocular tumour and others.

Of these, five ocular diseases, including lens disorders, vitreous/retina diseases, ocular trauma, glaucoma and strabismus, included more than 5% of the total number of patients and were calculated to statistical significance. The multivariate logistic regression model was used to estimate the risk factors for HBV and TP.

All statistical analyses were performed using the Statistical Package for the Social Sciences, version 16.0 (SPSS, Inc., Chicago, USA). The accepted level of significance for all analyses was *P*<0.05. For HBV and TP analysis, those aged below 20 years were considered as the reference, because the HBV vaccine was available in China 20 years ago and the population below 20 years had little opportunity for sexual contact in China. Lens diseases were regarded as a reference as they represented the major ocular disease in our study and included nearly half of the total participants.

## Results

### Characteristics of the Eye Patients

**Figure 1:**
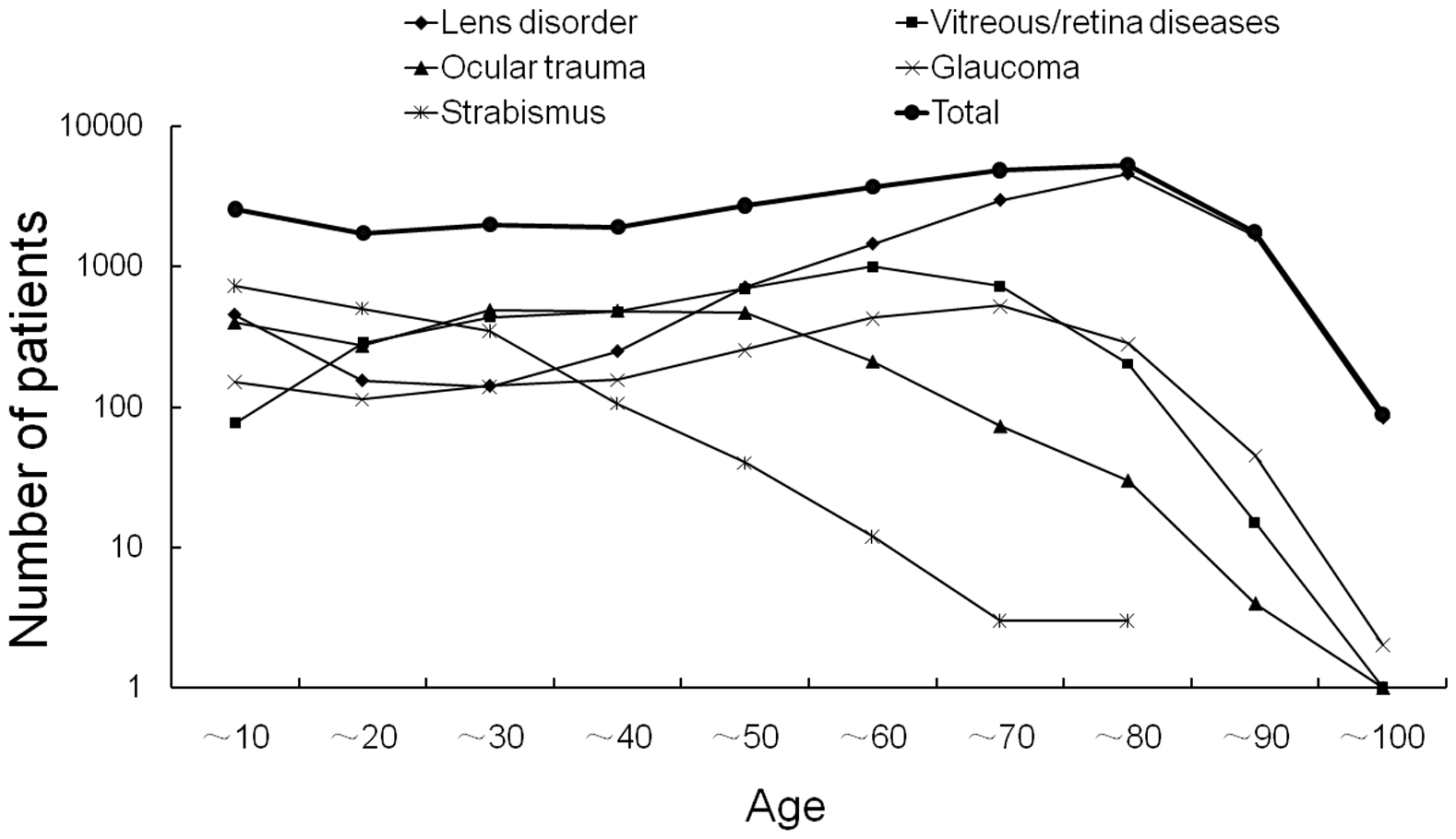
Demographic profile of the eye patients by age group. Statistics for the total and five major ocular diseases, including lens disorders, vitreous/retina diseases, glaucoma, ocular trauma and strabismus, which included more than 5% of the total patients in the various age groups were calculated.

The study recruited a total of 26,386 inpatients and/or surgery patients (male, 13,904, 52.7%; female, 12,482, 47.3%), who were tested the first time they came to the hospital. The age distributions of these diseases are shown in Figure 1. In general, our patients revealed uneven distributions among all age groups. Five categories of ocular diseases, i.e., lens disorders, vitreous/retina diseases, trauma, glaucoma and strabismus, accounted for more than 5% of the total patients. Further statistical analyses were performed on these five major ocular diseases.

### Prevalence of HBV, HCV, TP and HIV

**Figure 2:**
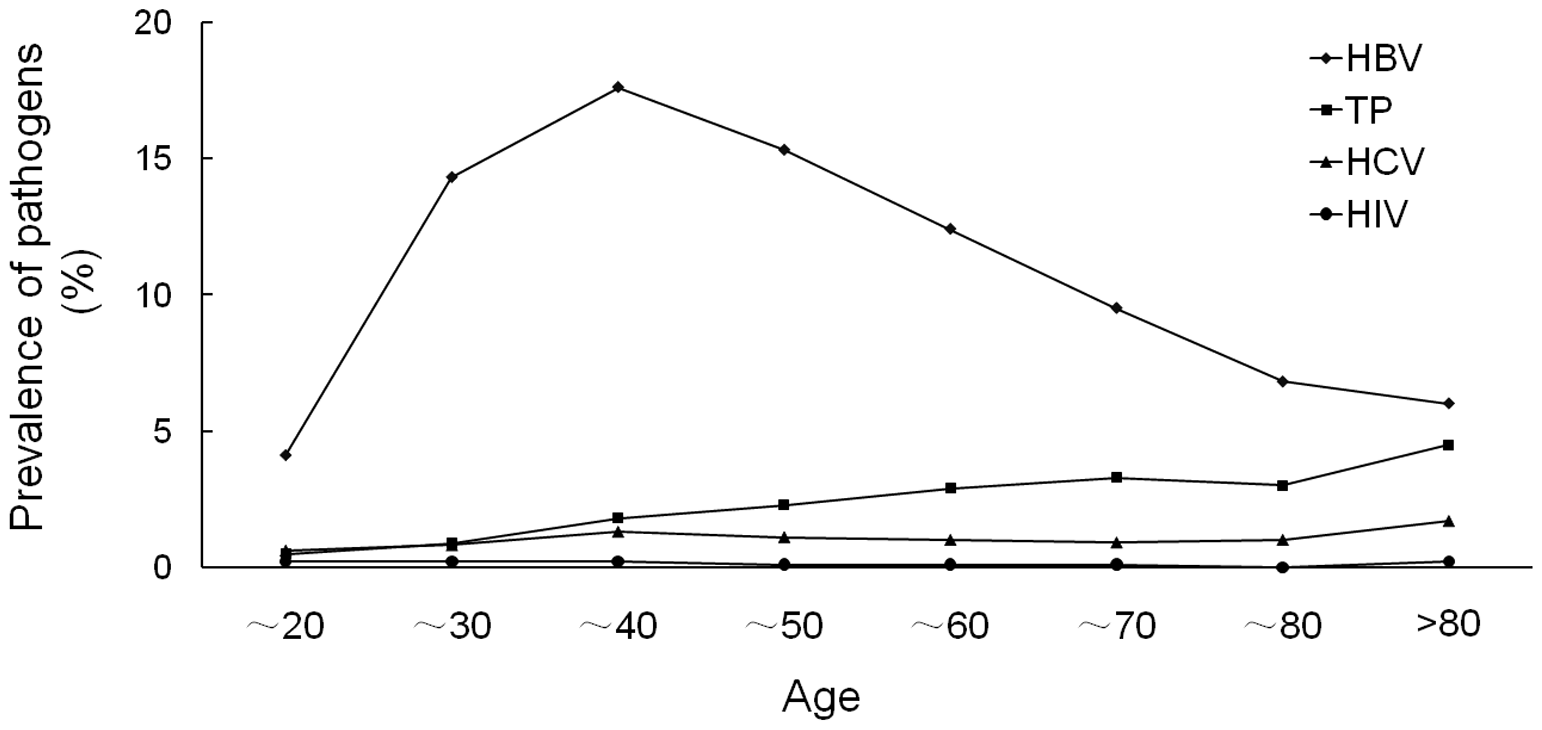
HBV, HCV, HIV and TP seroprevalence among eye patients by age groups.

The overall prevalence of HBV, HCV, TP and HIV was 9.79% (2583/26386), 0.99% (261/26385), 2.43% (642/26385) and 0.11% (30/26379), respectively. The prevalence of these pathogens in various age groups is presented in Table 1 and Figure 2. They show that there is a varied prevalence for the four pathogens in the different age groups of eye patients. The age and gender distributions were significantly different between HBV- positive and -negative cases (p<0.001). The prevalence of TP and HCV was different in the various age groups between positive and negative patients (p< = 0.001). There were no significant differences for HIV infection since there were only a few positive cases in our eye patients. Moreover, 186 subjects were co-infected with two or three pathogens. Among them, 15 for co-infection of HBV and HIV, 89 for co-infection of HBV and TP, 37 for co-infection of HCV and HBV, 25 for co-infection of HCV and TP, 4 for co-infection of HIV and TP, 1 for co-infection of HCV and HIV, 2 for co-infection of HBV and HIV and TP, 13 for co-infection of HBV and HCV and TP.

**Table 1:** Age, gender and category of eye patients*.

Variable	HBV(+)	HBV(−)	TP(+)	TP(−)	HCV(+)	HCV(−)	HIV(+)	HIV(−)
	N (%)	N (%)	N (%)	N (%)	N (%)	N (%)	N (%)	N (%)
Age (years) 0–20	175(6.8)	4086(17.2)	21(3.3)	4239(16.5)	26(10.0)	4234(16.2)	6(20.0)	4253(16.1)
21–30	284(11.0)	1696(7.1)	18(2.8)	1962(7.6)	15(5.7)	1965(7.5)	3(10.0)	1976(7.5)
31–40	334(12.9)	1565(6.6)	35(5.5)	1864(7.2)	25(9.6)	1874(7.2)	4(13.3)	1894(7.2)
41–50	412(16.0)	2288(9.6)	63(9.8)	2637(10.2)	30(11.5)	2670(10.2)	2(6.7)	2697(10.2)
51–60	456(17.7)	3215(13.5)	108(16.8)	3563(13.8)	35(13.4)	3636(13.9)	5(16.7)	3666(13.9)
61–70	456(17.7)	4340(18.2)	160(24.9)	4636(18.0)	45(17.2)	4751(18.2)	5(16.7)	4790(18.2)
71–80	355(13.7)	4885(20.5)	155(24.1)	5085(19.8)	51(19.5)	5189(19.9)	2(6.7)	5237(19.9)
81–	111(4.0)	1728(7.3)	82(12.8)	1757(6.8)	34(13.0)	1805(6.9)	3(10.0)	1836(7.0)
	*P*<0.001		*P*<0.001		*P* = 0.001		*P* = 0.419	
Gender Male	1545(59.8)	12359(51.9)	348(54.2)	13556(52.7)	128(49.0)	13776(52.7)	20(66.7)	13882(52.7)
Female	1038(40.2)	11444(48.1)	294(45.8)	12187(47.3)	133(51.0)	12348(47.3)	10(33.3)	12467(47.3)
	*P*<0.001		*P* = 0.438		*P* = 0.235		*P* = 0.125	
Category Lens disorder	1034(40.0)	11437(48.0)	390(60.7)	12081(46.9)	140(53.6)	12331(47.2)	11(36.7)	12458(47.3)
Vitreous/retina diseases	462(17.9)	3423(14.40	84(13.1)	3801(14.8)	42(16.1)	3843(14.7)	6(20.0)	3879(14.7)
Ocular trauma	355(13.7)	2084(8.8)	41(6.4)	2398(9.3)	20(7.7)	2419(9.3)	4(13.3)	2434(9.2)
Glaucoma	246(9.5)	1845(7.8)	59(9.2)	2032(7.9)	20(7.7)	2071(7.9)	5(16.7)	2085(7.9)
Uveitis	15(0.6)	114(0.5)	2(0.3)	127(0.5)	1(0.4)	128(0.5)	0(0.0)	129(0.5)
Cornea diseases	103(4.0)	851(3.6)	13(2.0)	941(3.7)	6(2.3)	948(3.6)	0(0.0)	954(3.6)
Orbital diseases	18(0.7)	130(0.5)	3(0.5)	145(0.6)	0(0.0)	148(0.6)	0(0.0)	148(0.6)
Lacrimal duct diseases	58(2.2)	411(1.7)	9(1.4)	460(1.8)	4(1.5)	465(1.8)	0(0.0)	468(1.8)
Strabismus	113(4.4)	1633(6.9)	15(2.3)	1731(6.7)	16(6.1)	1730(6.6)	3(10.0)	1742(6.6)
Oculoplastics	76(2.9)	900(3.8)	12(1.9)	963(3.7)	6(2.3)	969(3.7)	0(0.0)	975(3.7)
Oncology	76(2.9)	780(3.3)	10(1.6)	846(3.3)	3(1.1)	853(3.3)	1(3.3)	855(3.2)
Optic nerve diseases	23(0.9)	172(0.7)	4(0.6)	191(0.7)	3(1.1)	192(0.7)	0(0.0)	195(0.7)
Else	4(0.2)	23(0.1)	0(0.0)	27(0.1)	0(0.0)	27(0.1)	0(0.0)	27(0.1)
	*P*<0.001		*P*<0.001		*P* = 0.224		*P* = 0.655	

* 26386 patients were tested for HBsAg, 26385 for anti-HCV and TP, 26379 for anti-HIV.

The prevalence of HBV, TP, HCV and HIV in the different ocular disease categories was calculated (Table 1). Both HBV and TP infections revealed significant differences among various ocular diseases (p<0.001). As the prevalence of HCV and HIV was less than 1% of the total patients, we chose HBV and TP for logistic regression analysis to investigate the relationship between their infection and the five major ocular diseases.

### Characteristics of HBV Prevalence in Eye Patients

HBV prevalence in the different age groups is shown in Figure 2. Age-specific prevalence was 4.1% among those aged 1 to 20 years and 14.3% among those aged 21 to 30 years. It peaked in 31 to 40 year old patients (17.6%). After 40 years, the prevalence of HBV infection decreased gradually to 6.0% in patients older than 80 years. With regards to gender differences, males had a significantly higher prevalence (11.1%) than females (8.3%) in eye patients.

Using univariate logistic regression analysis, we identified the risk factors associated with being an HBV carrier in the different age groups, by gender and by the ocular disease categories (Table 2). Compared to patients younger than 20 years, there was a significant difference among the other age groups of eye patients. Also, female eye patients had a lower risk of HBV infection (OR: 0.726). In addition, compared with patients with lens diseases, patients with vitreous/retina diseases (OR: 1.493), trauma (OR: 1.884) and glaucoma (OR: 1.475) had a higher risk while patients with strabismus had a lower risk (OR: 0.765) of HBV infection.

**Table 2:** Univariable logistic regression analysis of HBV prevalence.

Variable	Category	OR	95% CI for *OR*		P value
			Lower	Upper	
Age (years)	0–20	Ref	–	–	Ref
	21–30	3.910	3.212	4.760	<0.001
	31–40	4.983	4.113	6.038	<0.001
	41–50	4.204	3.497	5.054	<0.001
	51–60	3.312	2.765	3.966	<0.001
	61–70	2.453	2.050	2.935	<0.001
	71–80	1.697	1.409	2.043	<0.001
	81–	1.500	1.175	1.915	0.001
Gender	Males	Ref	–	–	Ref
	Females	0.726	0.668	0.788	<0.001
Eye diseases	Lens disorder	Ref	–	–	Ref
	Vitreous/retina diseases	1.493	1.329	1.677	<0.001
	Ocular trauma	1.884	1.656	2.144	<0.001
	Glaucoma	1.475	1.273	1.709	<0.001
	Strabismus	0.765	0.626	0.936	0.009

Furthermore, using multivariable analysis adjusted for age and gender, the risk factors associated with HBV infection and their 95% CI were analysed for the different ocular diseases (Table 3). The results revealed that only trauma (OR: 1.332) and glaucoma (OR: 1.211) patients had a higher risk of HBV infection than those with lens diseases.

**Table 3:** Multivariate logistic regression of HBV prevalence adjusted by age and gender.

Eye diseases	*OR* § *_adj_*	95% CI for *OR_adj_*		P value
		Lower	Upper	
Lens disorder	Ref	–	–	
Vitreous/retinadiseases	1.052	0.922	1.199	0.452
Ocular trauma	1.332	1.136	1.563	<0.001
Glaucoma	1.211	1.038	1.414	0.015
Strabismus	0.978	0.769	1.245	0.858

§ age and gender were adjusted.

### Characteristics of TP Prevalence in Eye Patients

The trend of TP prevalence increased gradually with age (Figure 2), with 0.5% infected among those younger than 20 years to 4.5% infected among those older than 80 years. There were no significant differences based on gender.

The prevalence of TP was analysed by logistic regression analysis. Firstly, using univariate logistic regression analysis, the risk factors for TP in the various age groups, by gender and by ocular diseases, were identified (Table 4). Compared with patients younger than 20 years, those older than 30 years had a higher risk of TP infection. Compared to patients with lens diseases, those with vitreous/retinal diseases (OR: 0.685), trauma (OR: 0.530) and strabismus (OR: 0.268) had a lower risk of TP infection. However, by the multivariable analysis adjusted for age and gender, there were no significant differences among ocular diseases (Table 5).

**Table 4:** Univariable logistic regression analysis of TP prevalence.

Variable	Category	OR	95% CI for *OR*		P value
			Lower	Upper	
Age (years)	0–20	Ref	–	–	Ref
	21–30	1.852	0.985	3.484	0.056
	31–40	3.790	2.201	6.528	<0.001
	41–50	4.823	2.936	7.921	<0.001
	51–60	6.119	3.826	9.785	<0.001
	61–70	6.967	4.412	11.000	<0.001
	71–80	6.153	3.894	9.723	<0.001
	81–	9.421	5.814	15.264	<0.001
Gender	Males	Ref	–	–	Ref
	Females	0.940	0.803	1.100	0.438
Eye diseases	Lens disorder	Ref	–	–	Ref
	Vitreous/retina diseases	0.685	0.539	0.869	0.002
	Ocular trauma	0.530	0.383	0.733	<0.001
	Glaucoma	0.899	0.681	1.187	0.455
	Strabismus	0.268	0.160	0.451	<0.001

**Table 5:** Multivariate logistic regression of TP prevalence adjusted by age and gender.

Eye diseases	*OR* § *_adj_*	95% CI for *OR_adj_*		P value
		Lower	Upper	
Lens disorder	Ref	–	–	
Vitreous/retinadiseases	0.875	0.671	1.139	0.320
Ocular trauma	1.004	0.687	1.469	0.982
Glaucoma	1.104	0.827	1.473	0.502
Strabismus	1.190	0.635	2.230	0.588

§ age and gender were adjusted.

## Discussion

Testing for blood-borne pathogens is a fundamental and important procedure in preventing the transmission of pathogens from patient to HCP and vice versa and from patient to patient. This report provides an overview of the seroprevalence of HIV, HBV, HCV and TP among eye patients and is intended to increase the awareness of the transmission of blood-borne pathogens in a variety of ophthalmological practices and to analyse the relationship between their infection and various ocular diseases.

Our data showed that the overall seroprevalence of HBV, HCV, TP and HIV was 9.79%, 0.99%, 2.43% and 0.11%, respectively. Compared with previous studies with voluntary blood donors [10], [12], our study found a higher prevalence of HBV, HCV, TP and HIV. The blood donors were young adults and could have been aware of their own health status before they volunteered to donate blood.

In the current study, we identified several factors impacting the prevalence of HBV in eye patients. These were: being an adult older than 20 years, being male and having ocular trauma or glaucoma. Starting in 1992, the Chinese Ministry of Health recommended the routine immunisation of infants with the hepatitis B vaccine. Until 2002, the infant hepatitis B vaccination was added to China’s National Immunization Programme; the hepatitis B vaccine became free to all infants in 2005 [28]. Our study showed that overall prevalence of HBV was 9.79% in all eye patients and a dramatically low incidence was observed among patients younger than 20 years (4.1%). In multi-stage random study of 1967 children younger than 15 years in the Guangdong Province (the same area as our study), the authors revealed that the prevalence rate of HBsAg decreased from 19.86% in the 1992 survey to 4.91% in the 2006 survey [29]. We are gratified to see that patients younger than 20 years old had a significantly lower prevalence of HBsAg than those in other age groups because of introduction of the HBV vaccination program.

In addition to hepatitis B, hepatitis C is another major and serious global public health problem, which can cause liver cirrhosis and liver cancer with chronic consequences. Our data revealed that the seroprevalence of HCV was 0.99% in eye patients, which was similar to the 0.9% found by a survey of 8,226 residents between 25 and 65 years old in Anyang, Henan [30]. HCV infection in blood donors has decreased in recent years [10], [12]. However, it has been reported that the transmission of HCV occurs primarily through large or repeated direct percutaneous exposures to blood and that HCV is on average six times more likely than HIV to be transmitted after a percutaneous exposure [31]. Currently, occupational HCV transmission is only preventable through prevention of blood exposure. Therefore, it is important for HCP to be aware of preventing HCV in hospitals.

In the present study, by detecting a positive reaction to anti-TP, we found that the overall seroprevalence of TP was 2.43%. In addition, the prevalence increased with age from 0.5% among those younger than 20 years to 4.5% among those older than 80 years and a high risk of TP was associated with patients older than 30 years (Table 4). Epidemical information of syphilis in the general population in China is very rare. Several studies had found a higher prevalence of TP among the high-risk population, such as men who have sex with men [32] and female sex workers [33]. Evaluating the prevalence and incidence of serological markers for TP among 801,511 blood donors from five Chinese blood centres, Liu et al. reported that the presence of higher TP markers was associated with donors older than 25 years [34]. In our study, eye patients displayed an increasing seroprevalence from childhood to 80 years old. Since TP is mainly transmitted by close contact, these results may highlight our preventive strategies for TP in the future.

The estimate of risk for HIV transmission after exposure to infected blood is approximately 0.3% [35]. Meanwhile, till 2001 in the United States, there were 57 documented cases of occupational transmission of HIV after exposure to a patient’s blood or body fluids in HCP [36]. In hospital care settings, HIV transmission is limited by preventing exposures to blood, especially percutaneous injuries. Among our 26,368 eye patients, 30 (0.11%) were infected with HIV, which was diagnosed by a chemiluminescence immunoassay and Western blot analysis. In our hospital, extra care was taken with these patients when they were operated on and during their stay; these cost a lot more than taking care of patients without HIV infection.

A very interesting finding in the present study is that, compared to lens diseases, ocular trauma (OR: 1.332) and glaucoma (OR: 1.211) were considered to be risk factors associated with HBV infection, when analysed by the multivariate logistic regression model. In other words, there is a high risk of HBV infection in ocular trauma and glaucoma patients. It has been reported that occupation was the highest risk factor influencing HBV infection. Peasants, labourers and private, small-businessmen had a higher risk of HBV infection compared with white-collar professionals [37]. Furthermore, special populations, such as long-distance truck drivers, construction workers and migrant workers, contributed to the high prevalence of HBV [38]. It is reasonable that our ocular trauma patients, who were mainly composed of male workers, had a high HBV sereprevalence. However, glaucoma patients displayed a higher seroprevalence of HBV when compared to patients with lens diseases after age and gender correction. There have been two reported cases that indicated that hepatitis B patients on interferon therapy developed glaucoma [39], [40]. Our eye patients did not receive interferon therapy. The reasons for our glaucoma patients having a high seroprevalence of HBsAg remain to be clarified.

Studies on the prevalence of blood-borne pathogen infections in different patients, such as those with chronic renal failure [18], myeloma patients [19], those with sickle cell diseases [41] and those with mental diseases [42] have been studied. To our knowledge, this is the first report to determine the infection status of four pathogens in eye patients. This report provides information that ocular professionals can use to prevent infections during exposure prone procedures.

In summary, a total of 26,386 eye patients who needed operations and/or hospitalization were screened for HBV, HCV, HIV and TP. The seroprevalence of these four pathogens were reported in patients with different ocular diseases. There were high levels of HBV in patients with ocular trauma and glaucoma compared to those with lens diseases. This knowledge on pathogen infection of eye patients reminds HCP at risk for exposure to potentially infected blood and other body fluids and for repeated direct cutaneous and mucous membrane contacts to be careful. Measures aimed at preventing exposure to pathogens, including pre-operation screening, should be consistently utilised during medical procedures.
